# The predictive value of MR cytometry in histological differentiation of rectal cancer: an exploratory study

**DOI:** 10.1007/s00330-026-12341-w

**Published:** 2026-01-30

**Authors:** Qing Zhao, Diwei Shi, Hongxia Zhong, Chaoyang Jin, Zongshu Wang, Zhuo Shi, Lin Li, Bingjing Wang, Yueluan Jiang, Thorsten Feiweier, Junzhong Xu, Hua Guo, Hongmei Zhang

**Affiliations:** 1 Department of Diagnostic Radiology, National Cancer Center/National Clinical Research Center for Cancer/Cancer Hospital, Chinese Academy of Medical Sciences and Peking Union Medical College, Beijing, China; 2 Department of Radiology, Shanxi Province Cancer Hospital/Shanxi Hospital Affiliated to Cancer Hospital, Chinese Academy of Medical Sciences/Cancer Hospital Affiliated to Shanxi Medical University, Taiyuan, China; 3 Research Institute of Tsinghua University in Shenzhen, Shenzhen, China; 4 Tsinghua Shenzhen International Graduate School, Tsinghua University, Shenzhen, China; 5 Vanderbilt University Institute of Imaging Science, Vanderbilt University Medical Center, Nashville, TN USA; 6 Center for Biomedical Imaging Research, School of Biomedical Engineering, Tsinghua University, Beijing, China; 7 Department of Pathology, National Cancer Center/National Clinical Research Center for Cancer/Cancer Hospital, Chinese Academy of Medical Sciences and Peking Union Medical College, Beijing, China; 8 School of Information Science and Technology, Beijing Forestry University, Beijing, China; 9 MR Research Collaboration Team, Siemens Healthineers Ltd, Beijing, China; 10 Research & Clinical Translation, MagneticResonance, Siemens Healthineers AG, Erlangen, Germany; 11 Department of Radiology and Radiological Sciences, Vanderbilt University Medical Center, Nashville, TN United States of America

**Keywords:** Diffusion-weighted imaging, MR cytometry, Rectal cancer, Microstructural imaging, Histological differentiation

## Abstract

**Purpose:**

To investigate and compare the diagnostic value of different MR cytometry methods in predicting histological differentiation of rectal tumors.

**Materials and methods:**

This prospective study (ClinicalTrials.gov identifier: NCT07107815) enrolled eligible patients with rectal cancer from March 2025 to July 2025. All patients underwent rectal MRI with oscillating gradient spin-echo and pulsed gradient spin-echo sequences. Microstructural parameters were obtained from three different MR cytometry methods. Based on pathological results, rectal tumors were classified as poor differentiation and well/moderate differentiation. Intergroup comparison was conducted using Mann–Whitney U-test. The diagnostic value of imaging metrics, including microstructural parameters and apparent diffusion coefficients (ADCs), in distinguishing rectal cancers with different histological differentiation was evaluated by logistic regression analysis.

**Results:**

A total of 86 patients were included (mean age: 60.5 ± 10.7 years; male proportion: 66.3%; maximal tumor diameter: 38.7 ± 11.1 mm), including 37 with poor differentiation, 49 with well/moderate differentiation. Intracellular volume fraction and cellularity were higher (*p* < 0.0001), while extracellular diffusivity, water exchange rate constant, and ADC metrics were lower (*p*-values from 0.01 to < 0.0001) in the poor-differentiation group. Among the classifiers based on a single imaging metric, intracellular volume fraction provided the highest areas under the receiver operating characteristic curves (AUC = 0.812). Clinical performance of the combined regression models incorporating microstructural parameters and ADC metrics (AUC = 0.883) was significantly superior to the conventional ADC measurement (AUC = 0.795).

**Conclusion:**

MR cytometry provides additional information over ADC measurements in identifying histological differentiation grades of rectal cancer; the integration of MR cytometry into clinical scans may improve the diagnostic performance of rectal MRI.

**Key Points:**

***Question*** *Microstructural parameters obtained from MR cytometry methods and apparent diffusion coefficients showed significant differences between rectal tumors with different histological differentiation grades.*

***Findings*** *The combined regression models, including both microstructural parameters and apparent diffusion coefficient metrics, provided a significantly higher value than the conventional PGSE-based ADC measurement.*

***Clinical relevance*** *Incorporating transcytolemmal water exchange into biophysical modeling can further improve the clinical performance over the impermeable model.*

**Graphical Abstract:**

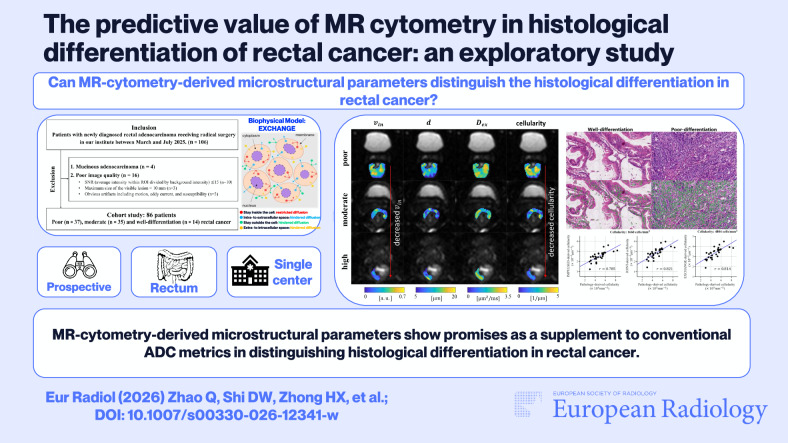

## Introduction

Colorectal cancer ranks third in all malignancies globally [1], where rectal cancer presents an increasing incidence in younger populations and still shows major challenges in diagnosis and treatment [2, 3]. Histological differentiation grades of rectal cancer before treatment are essential in designing individualized plans [4, 5], as poorly differentiated tumors typically exhibit poor prognosis, including local recurrence and regional lymph node metastasis, thus requiring more advanced therapeutic and monitoring methods. The histopathological examination of biopsy or surgical samples is the current approach for clinical assessment of histological differentiation grades for rectal cancer. However, the limitations of biopsy, including invasiveness, sampling biases of specimens, and time delays of surgical examination, all restrict its guiding significance in early individualized treatment of rectal cancer [6]. Therefore, developing a non-invasive method to accurately evaluate histological differentiation of rectal cancer before treatment holds practical significance.

Currently, pelvic MRI is a powerful complementary tool that addresses the inherent limitations of invasive methods and has become the most recommended imaging technique for locoregional risk factor evaluation of rectal cancer [7]. Diffusion-weighted MR imaging (dMRI) also demonstrated superior performance in particular clinical tasks, such as distinguishing between tumors and fibrotic reactions and gaining insights into the heterogeneity of the microstructure within tumors, through measuring water diffusion [7, 8]. However, conventional dMRI-obtained metrics characterize the overall properties of microstructures within an imaging voxel, such as apparent diffusion coefficient (ADC), which measures the average diffusivity influenced by multiple pathological features simultaneously [9]. Their sensitivity to individual variations is typically poor [10].

Recently, the time-dependence of diffusion-weighted signals has been investigated and used to detect microstructural features at different scales within in vivo tissues [11–14]. Specifically, water diffusion is restricted or hindered by tissue structures, as reflected in attenuated signals; the measured signals varies depending on the applied diffusion time ${t}_{{diff}}$, even if the final b-values are the same [15]. This ${t}_{{diff}}$-dependence has inspired us to capture microstructural information through introducing multiple-${t}_{{diff}}$ acquisitions in dMRI measurements. Such an imaging technology, MR cytometry, has been developed and shown promising potential in estimating microstructural features at cellular and even sub-cellular levels [10, 15, 16], which typically employs the joint protocol of pulsed gradient spin-echo (PGSE) and oscillating gradient spin-echo (OGSE) sequences [17, 18], to maximize the range of ${t}_{{diff}}$ and improve the sensitivity to different microstructural scales. A series of multi-compartmental models were proposed to decouple quantitative features contained in dMRI signals, such as IMPULSED (imaging microstructural parameters using limited spectrally edited diffusion) [13, 15], which simplifies cells as impermeable spheres and models signals arising from intra- and extracellular compartments. The feasibility of this concise but effective method has been evaluated in numerical simulations [13], in vitro cell experiments [13, 19], in vivo animal experiments [15]; and multiple clinical cancer studies, such as breast cancer [20–23], prostate cancer [24], gliomas [25, 26], and endometrial cancer [27].

However, the clinical value of MR cytometry remains unclear in the assessment of rectal cancer. The application of dMRI to hollow organs is prone to interference from intestinal gas, which may cause artifacts; furthermore, the irregular lesion shapes and intestinal peristalsis also pose difficulties in region of interest (ROI) delineation and slice-matching [28]. Although a preliminary study has explored the performance of IMPULSED in identifying benign/malignant rectal lesions [29], the investigation on histological differentiation grades remains limited, which is more difficult to identify through conventional MRI but crucial for individualized treatment. Therefore, we aim to evaluate the clinical performance of MR cytometry in distinguishing rectal cancers with different histological differentiation grades. Furthermore, in addition to the IMPULSED method, the recently proposed JOINT [19] and EXCHANGE [30], were also applied. These two emerging methods incorporated transcytolemmal water exchange into biophysical modeling to overcome the shortcomings of the “impermeable” assumption, resulting in more accurate microstructural restoration and an additional parameter for cell membrane permeability. The comparison between the three methods demonstrated the clinical significance of introducing transcytolemmal water exchange into MR cytometry, providing valuable insights for future studies on rectal cancer.

## Materials and methods

### Patients

**Fig. 1:**
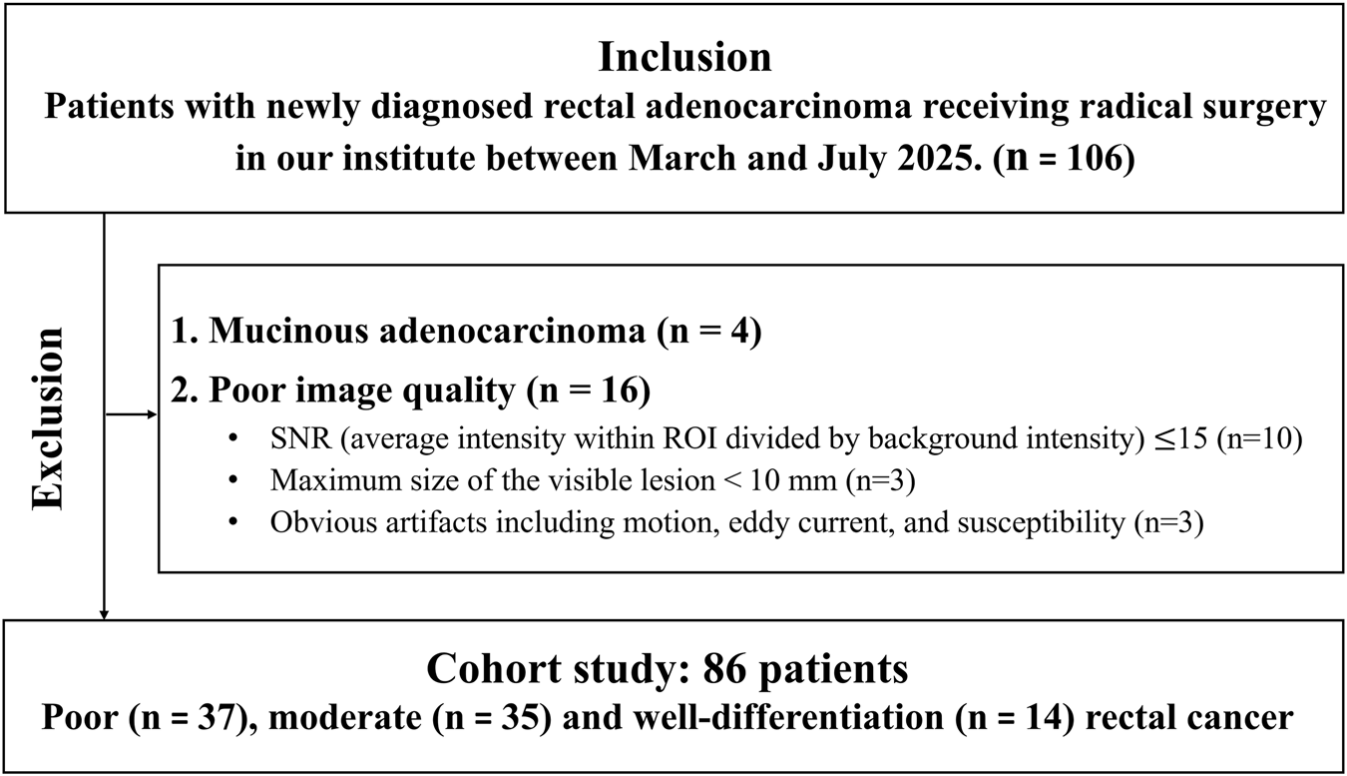
Flowchart of participant enrollment

This prospective study (ClinicalTrials.gov identifier: NCT07107815) has been approved by the ethics committee of our hospital; written informed consent was received from all participants. The inclusion criteria were: (1) newly biopsy-diagnosed rectal adenocarcinoma without previous treatment; (2) complete radical surgery in our institute and signing the informed consent form; (3) underwent rectal MRI (without any preoperative treatment) within 2 weeks before surgery. The exclusion criteria were: (1) inadequate MR image quality for analysis; (2) incomplete clinicopathological data; (3) excessive mucinous component in subsequent pathology. In total, 106 eligible patients were recruited between March and July 2025. Among them, 4 cases were excluded due to large amounts of mucinous component; and 16 cases were excluded due to poor image quality with signal to noise ratios (SNR, calculated by dividing the average signal intensity within the ROI by the background signal intensity) less than 15 (*n* = 10), the maximal diameter of the lesion on high-resolution T2-wighted images (HR-T2WI) less than 10 mm (*n* = 3), and image artifacts, such as motion, eddy current, and susceptibility artifacts (*n* = 3). Furthermore, it should be noted that given the low proportion (10–20%) of poorly-differentiated rectal cancers, we adopted a targeted, non-consecutive enrollment approach through prospectively screening patients’ prior colonoscopy biopsy pathology reports. Specifically, we prioritize recruiting patients with biopsy-confirmed poor differentiation and schedule rectal MRI examinations at least 2 weeks prior to surgery to maximize patient enrollment efficiency. Finally, 86 participants (mean age: 60.5 $\pm$ 10.7 years; male proportion: 66.3%; maximal tumor diameter: 38.7 ± 11.1 mm) were enrolled in the cohort study (Table 1) with 37 poor differentiation (43%) and 49 well/moderate differentiation. The corresponding patient enrollment workflow chart is shown in Fig. 1.

**Table 1:** Patient information and lesion characteristics

Grouping	Poor-differentiation	Well/moderate differentiation		*p*-value
Number	Poor differentiation *n* = 37	Moderate differentiation *n* = 35	Well differentiation *n* = 14	
Age (years)	60.4 ± 11.7	60.5 ± 10.7	60.8 ± 5.3	0.299
Gender (male: female)	25:12	23:12	9:5	0.827
CEA level (ng/mL) Median (min, max)	4.74 (0.95, 181)	4.78 (1.01, 135)	2.95 (2.05, 128)	0.145
CA 19-9 level (U/mL) Median (min, max)	19.5 (< 2*, 666)	12.2 (< 2, 207)	12.4 (< 2, 119)	0.063
Maximal diameter (mm)	40.4 ± 12.2	37.1 ± 10.5	37.4 ± 9.5	0.765
Tumor location				
Upper segment	10	9	4	0.879
Middle segment	21	19	8	
Lower segment	6	7	2	
pT stage				
pT_2_	3	2	3	0.927
pT_3_	28	27	10	
pT4a	6	6	1	
pN stage				
pN0	12	17	7	0.292
pN1	17	10	6	
pN2	8	8	1	

When the serum level of CA 19-9 is less than 2 U/mL, the specific value cannot be measured and is simply indicated as “< 2”

All examination results were obtained from baseline (pre-treatment)

*CEA* carcinoembryonic antigen, *CA 19-9* carbohydrate Antigen 19-9, *EMVI* extramural vascular invasion

### MRI acquisition

Rectal dMRI examinations were performed on a 3-T MR scanner (MAGNETOM Prisma, Siemens Healthineers AG) with maximum gradient amplitude of 80 mT/m and maximum slew rate of 200 T/m/s, using an 18-channel body coil. The MR cytometry acquisition contained two trapezoidal cosine-modulated OGSE sequences with the oscillating frequencies of 25 Hz and 50 Hz and one conventional PGSE sequence. Other conventional rectal MRI sequences were acquired simultaneously. The detailed MRI protocol is shown in Table 2.

**Table 2:** Rectal MRI protocol

Parameter	T1WI	High-resolution T2WI	Contract-enhanced T1WI	Diffusion-weighted images		
				OGSE_25HZ_	OGSE_50HZ_	PGSE
TR (ms)	522	5410	2.99	3300		
TE (ms)	9.1	104	1.21	121		
FOV (mm^2^)	360 × 360	240 × 240	260 × 260	380 × 348		
Voxel size (mm^3^)	0.9 × 0.9 × 6	0.5 × 0.5 × 3	1.1 × 1.1 × 2	1.6 × 1.6 × 4		
Slices	24	24	72	12		
Bandwidth (Hz/pixel)	303	200	590	2492		
b-values (s/mm²)	-	-	-	0, 250, 500, 750, 1000	0, 250, 500	0, 250, 500, 750, 1000
${{{\rm{\delta }}}}/\Delta$ (msec)	-	-	-	41.52/60	41.52/50	13.52/74
Cycles	-	-	-	1	2	-
${t}_{{diff}}$ (msec)	-	-	-	10	5	69.5
Scanning time	1′34″	2′59″	2′41″	2′09″	53″	1′59″

${{{\rm{\delta }}}}$ is the duration of each diffusion gradient, $\Delta$ is the separation of two diffusion gradients, $f$ is the oscillating frequency of diffusion gradients, ${t}_{{diff}}$ is the diffusion time. For PGSE,$\,{t}_{{diff}}\,=\Delta -{{{\rm{\delta }}}}/3$, while for OGSE, empirically ${t}_{d}=1/(4f)$

*PGSE* pulsed gradient spin-echo, *OGSE* oscillating gradient spin-echo, *T1WI* T1-weighted image, *T2WI* T2-weighted image, *DWI* diffusion-weighted image, *TR* repetition time, *TE* echo time, *FOV* field of view

### Data processing

Image registration among the three sequences, i.e., PGSE, OGSE-25Hz, and OGSE-50Hz, was performed in the MATLAB R2022b software, using the built-in “imregtform” and “imwarp” functions. The images from OGSE-25Hz were employed as the alignment template because this sequence was acquired between OGSE-50Hz and PGSE. Using the image from the intermediate sequence as the template may improve spatial matching. In addition, Marchenko-Pastur principal component analysis was performed to achieve the four-dimensional MRI (length, width, height, and b-value) denoising. This method is based on the data redundancy in the principal component domain using the universal properties of the eigen-spectrum of random covariance matrices [31]. Finally, the intravoxel incoherent motion (IVIM) effect was removed by recalculating the non-diffusion-weighted signals. Specifically, a log-linear fitting was performed for the signals of $200 < b < 1000$ s/mm^2^ to obtain the revised raw signals ($b=0$ s/mm^2^) [10].

### Regions of Interest

Images were loaded into ITK-SNAP software (Version 4.0, www.itksnap.org). The regions of interest (ROIs) were drawn by two experienced radiologists with 12 (Q.Z.) and 8 (H.X.Z.) years of experience, respectively, blinded to the clinicopathological information. The radiologists manually drew the ROIs on the diffusion-weighted images of OGSE 25 Hz, while referencing the images of HR-T2WI. Surrounding fat, cavity, mucinous and necrotic components were excluded from the ROIs, and the lesion on all tumor-visible slices has been carefully delineated. The Dice similarity coefficient between ROIs from two radiologists was 0.794 ± 0.059 for all 86 patient cases. The final ROIs were resolved by consensus. The maximal tumor diameter and tumor location were evaluated as previously reported [32].

### Data analysis in MR cytometry

Three MR cytometry methods, including IMPULSED, JOINT, and EXCHANGE, were implemented to analyze diffusion images from the OGSE and PGSE acquisitions.

## Impulsed

**Fig. 2:**
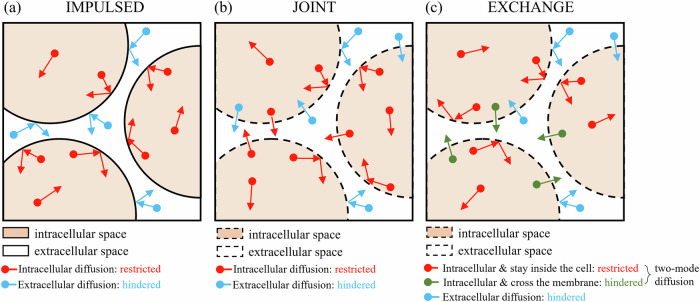
Tumor tissue modeling in the three MR cytometry methods. **a** IMPULSED: tumor cells are modeled as impermeable cells. **b** JOINT: transcytolemmal water exchange was incorporated to enhance the characterization of restricted/hindered diffusion. **c** EXCHANGE: the intracellular water molecules are further characterized by the two-mode diffusion

This method models the acquired signals $S$ as the sum of signals arising from the intra- (${S}_{{in}}$) and extracellular (${S}_{{ex}}$) compartments, and transcytolemmal water exchange is ignored (Fig. 2a):

1 $$S={v}_{{in}}{S}_{{in}}+\left(1-{v}_{{in}}\right){S}_{{ex}},$$

where ${v}_{{in}}$ is the intracellular volume fraction. The intracellular signal ${S}_{{in}}$ equals $\exp (-b\cdot {AD}{C}_{r})$, where the apparent restricted diffusion coefficient ${AD}{C}_{r}$ is a function of the intracellular intrinsic diffusivity ${D}_{{in}}$ and cell diameter $d$, its analytical expressions have been derived previously (as shown in Appendix S1). The extracellular signal ${S}_{{ex}}$ equals to $\exp (-b\cdot {D}_{{ex}})$, where ${D}_{{ex}}$ is a constant extracellular diffusivity.

## Joint

This emerging method incorporates water exchange between the intra- and extracellular compartments into the IMPULSED framework (Fig. 2b). For the PGSE acquisitions, the original analytical expression of dMRI signals in IMPULSED is inserted into the Kärger model, which can be expressed as:

2 $$S={V}_{1}\exp \left(-b{D}_{1}^{* }\right)+\left(1-{V}_{1}\right)\exp \left(-b{D}_{2}^{* }\right),$$

where the variables ${V}_{1}$, ${D}_{1}^{* }$ and ${D}_{2}^{* }$ are functions of the microstructural parameters $d$, ${v}_{{in}}$,${D}_{{in}}$, ${D}_{{ex}}$ and water exchange rate constant ${k}_{{in}}$. Their derivations are provided in Appendix S2. For the OGSE signals, JOINT assumes that the impact of water exchange is limited, and the analytical expressions remain consistent with IMPULSED.

## Exchange

This method also combines the IMPULSED framework and the Kärger model, but comprehensively considers the impact of water exchange, not only for PGSE but also for OGSE. Specifically, a dual-mode diffusion model is proposed to describe the actual water diffusion within cells, which includes both restricted and hindered diffusion in the presence of transcytolemmal water exchange (Fig. 2c); a dimension-based expression is used to correct for the edge-enhancement effect induced by restrictions; a discretization computational framework is proposed to adapt this method to arbitrary gradient waveforms. More details are shown in Appendix S3.

### Data fitting

For all three methods, the microstructural parameters were calculated on the data fitting platform-MATI [33]. There are three free parameters in IMPULSED: ${v}_{{in}}$, $d$, and ${D}_{{ex}}$. And for JOINT and EXCHANGE, an additional free parameter ${k}_{{in}}$ is introduced into biophysical modeling. The image-derived “cellularity” $\rho$ was calculated as: ${v}_{{in}}/d\times 100$ [24]. The intracellular intrinsic diffusivity ${D}_{{in}}$ was fixed as 1.56 ${{{\rm{\mu }}}}{{{{\rm{m}}}}}^{2}/{{{\rm{ms}}}}$ in to stabilize the model fitting as reported previously [20]. The time-dependent ADC metrics were obtained by fitting the multi-*b*-value signals to $S=\exp (-b\cdot {{{\rm{ADC}}}})$.

### Pathological analysis

The histopathological evaluation was achieved by staining the surgical specimens with hematoxylin and eosin (H&E). The histological analysis was performed by a pathologist with 12 years of experience (L.L.), who was blinded to the MRI examinations. The histological differentiation grades were assessed according to the latest World Health Organization criteria [34]: well-differentiated tumors were defined as having > 95% glandular structures, moderately differentiated tumors as having 50–95% glandular structures, and poorly-differentiated tumors as having 5–50% glandular structures. All dissected tumors and lymph nodes were sectioned and examined conventionally by H&E staining, and the TN stages were determined according to the 9th edition of the American Joint Committee on Cancer (AJCC) TNM classification.

For a comparative analysis of the radiological and pathological results, we have obtained the H&E-stained images of 31 included patients. The nuclei in H&E-stained images were segmented using CellPose, a generalist deep learning-based model pre-trained on diverse microscopy images encompassing various cell types and imaging conditions [35, 36]. The pathology-derived cellularity was calculated as $n/A$, where $n$ is the cell number identified by CellPose, and A is the area of the section. Subsequently, Pearson correlation analysis was performed to quantify the correlation between the image-derived cellularity (i.e., the parameter $\rho$) and above pathology-derived cellularity. A strong correlation (the correlation coefficient $r$ > 0.7) between them would support the rationality of MR cytometry imaging.

### Statistical analysis

The patients were classified into two groups: the poor-differentiation group and the well/moderate-differentiation group. The rank-sum test, i.e., Mann–Whitney U-test, was performed to evaluate the significance of differences between the imaging metrics obtained from the two groups. Logistic regression in the SPSS program was employed to evaluate the ability to identify rectal tumors with poor differentiation and well/moderate differentiation. The area under the receiver operating characteristic (ROC) curve (AUC) was computed to quantify the diagnostic value of each imaging metric. For the multivariable regression model, when the number of candidate variables exceeds four (in this study, the upper limit for the number of included variables was set to four, according to the sample size), variables were selected using a recently developed analysis method that combined Lasso and Ridge regressions [37, 38]. Briefly, we first include all variables in a combined regression model; then we set up a deviance function containing a penalty term; in the process of minimizing the deviance, we successively remove the variables with the corresponding regression coefficients of zero, until the number of remaining variables is reduced to four. The model coefficients, calibration slope/intercept/plot, and Brier score of all logistic regression models are shown in Table S1 and Fig. S2 in the Appendix. DeLong test was used to compare the differences between different logistic regression models.

## Results

### Imaging metrics mapping of rectal lesions

**Fig. 3:**
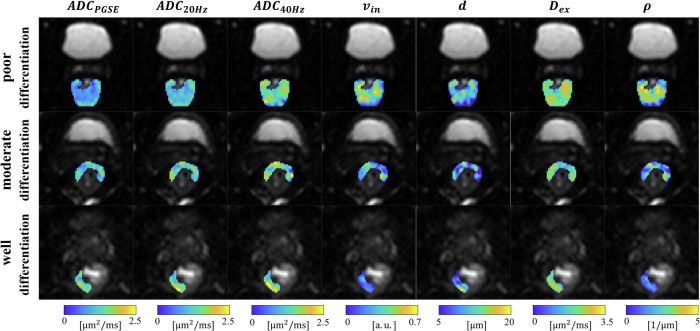
The imaging findings of three representative rectal tumors, with poor, moderate, and well differentiation grades, respectively. The background of all subplots is the PGSE image with b = 1000 s/mm^2^. The pseudo-color image displays the color-coded distribution of parameter values for a specific imaging metric, including three time-dependent ADCs, ${v}_{{in}}$, $d$, ${D}_{{ex}}$, and $\rho$, within the tumor lesion area (i.e., ROI). The plane of DWI is axial, and the hyperintense structure, especially visible on the maps with poor or moderate differentiation, is bladder. ADC, apparent diffusion coefficient; ${v}_{{in}}$, intracellular volume fraction; $d$, cell diameter; ${D}_{{ex}}$, extracellular diffusivity; $\rho$, image-derived cellularity; DWI, diffusion-weighted image; PGSE, pulsed gradient spin-echo

Figure 3 shows the maps of IMUPLSED-derived parameters and ADC metrics obtained from the representative rectal lesions with poor, moderate, and well differentiations, respectively. The ADC values in all three lesions increased as ${t}_{{diff}}$ decreases, i.e., ${{{\rm{AD}}}}{{{{\rm{C}}}}}_{{{{\rm{PGSE}}}}} < {{{\rm{AD}}}}{{{{\rm{C}}}}}_{25{{{\rm{Hz}}}}} < {{{\rm{AD}}}}{{{{\rm{C}}}}}_{50{{{\rm{Hz}}}}}$. For the microstructural parameters fitted by IMPULSED, the intracellular volume fraction ${v}_{{in}}$ and cellularity $\rho$ were higher in the tumor with poorer differentiation. In addition, the results of cell diameter $d$ and extracellular diffusivity ${D}_{{ex}}$ showed limited difference between the lesions with poor and well/moderate differentiations. The quantitative comparisons between the two groups are shown in the following sections.

### Intergroup comparison of imaging metrics

**Fig. 4:**
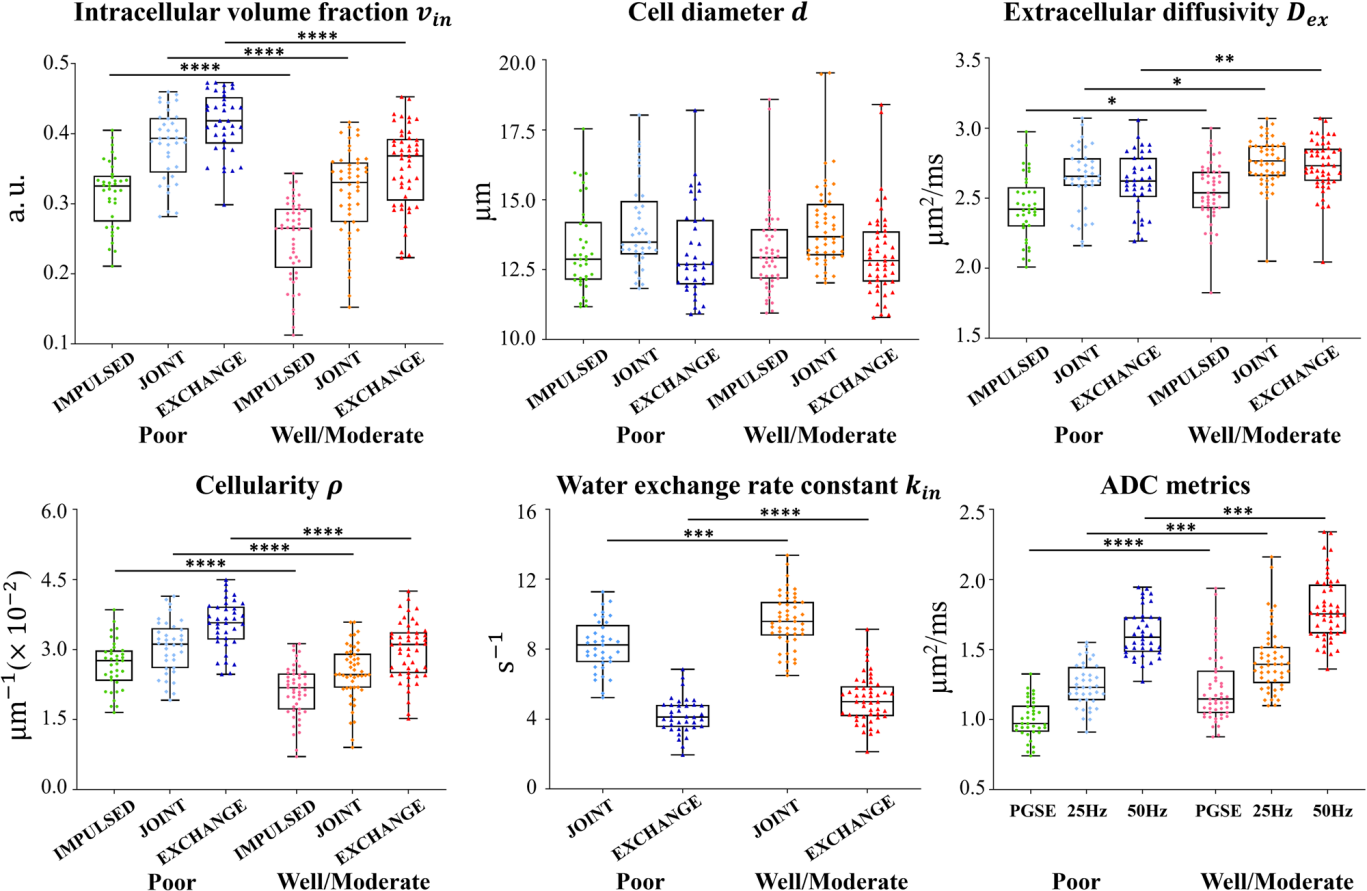
Intergroup comparison of ADC metrics and microstructural parameters between poorly-differentiated and well/moderately differentiated rectal tumors. * *p* < 0.05, ** *p* < 0.01, *** *p* < 0.001 and **** *p* < 0.0001

As shown in Fig. 4, all three ADC metrics, i.e., ${{{\rm{AD}}}}{{{{\rm{C}}}}}_{{{{\rm{PGSE}}}}}$, ${{{\rm{AD}}}}{{{{\rm{C}}}}}_{25{{{\rm{Hz}}}}}$ and ${{{\rm{AD}}}}{{{{\rm{C}}}}}_{50{{{\rm{Hz}}}}}$, were significantly lower in the poor-differentiation group. On the other hand, the comparison results on the microstructural parameters are essentially consistent for the three methods (IMPULSED, JOINT, and EXCHANGE). There was no significant difference in $d$, and $p$= 0.937, 0.882, and 0.896, respectively, for IMPULSED, JOINT, and EXCHANGE. The ${v}_{{in}}$ and $\rho$ values were both significantly higher in tumors with poor differentiation for all three methods. For ${D}_{{ex}}$ and ${k}_{{in}}$, the intergroup difference was also significant, with these two metrics being higher in the well/moderate-differentiation group. More detailed results regarding the intergroup comparison are shown in Appendix Table S2.

### Classification of lesions with poor- and well/moderate differentiation using imaging metrics

**Fig. 5:**
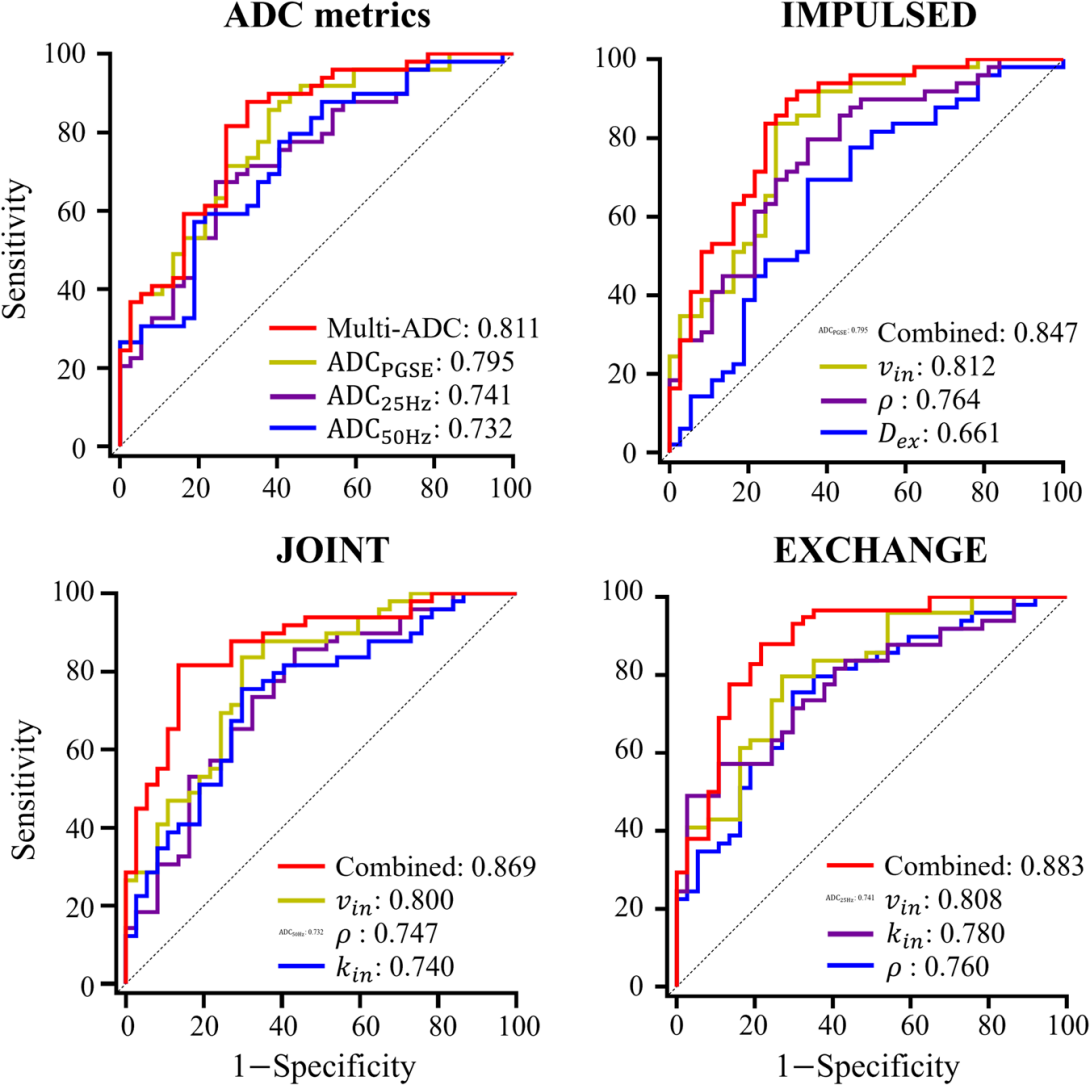
Receiver operating characteristic (ROC) curves of imaging metrics in discriminating poorly-differentiated and well/moderately differentiated rectal tumors. The numbers in the legend represent the areas under the ROC curves (AUC) of the regression models. Note that all ROC curves are from the same dataset. ADC, apparent diffusion coefficient; ${v}_{{in}}$, intracellular volume fraction; ${D}_{{ex}}$, extracellular diffusivity; $\rho$, image-derived cellularity; ${k}_{{in}}$, water exchange rate constant

As shown in Table 3 and Fig. 5, the ROC curve of ${{{\rm{AD}}}}{{{{\rm{C}}}}}_{{{{\rm{PGSE}}}}}$ provided the highest AUC of 0.795 among the classifiers based on a single ADC metric. And the AUC value can be further improved to 0.811 by combining the three different ADCs (multi-ADC). For the classifiers based on the microstructural parameters obtained from MR cytometry, the ROC curve of ${v}_{{in}}$ provided the highest AUCs for all three methods, with the values of 0.812, 0.800, and 0.809 for IMPULSED, JOINT, and EXCHANGE, respectively. Furthermore, the combination of microstructural parameters and ADC metrics has improved clinical performance, as evidenced by a higher AUC. Specifically, the combination of ${v}_{{in}}$, $d$, $\rho$, and ${{{\rm{AD}}}}{{{{\rm{C}}}}}_{25{{{\rm{Hz}}}}}$ improved the AUC to 0.847 for IMPULSED; the combination of ${D}_{{ex}}$, $\rho$, ${k}_{{in}}$, and ${{{\rm{AD}}}}{{{{\rm{C}}}}}_{50{{{\rm{Hz}}}}}$ improved the AUC to 0.869 for JOINT; the combination of $d$, $\rho$, ${k}_{{in}}$, and ${{{\rm{AD}}}}{{{{\rm{C}}}}}_{{{{\rm{PGSE}}}}}$ improved the AUC to 0.883 for EXCHANGE. In addition, compared to the diagnostic value of ${{{\rm{AD}}}}{{{{\rm{C}}}}}_{{{{\rm{PGSE}}}}}$ (corresponding to conventional DWI), the improvements obtained from the combined regression models based on the three methods (from 0.795 to 0.847, 0.869, and 0.883) are all significant for the Delong test. However, compared to the multi-ADC classifier (AUC = 0.811), the improvements are insignificant for IMPULSED (*p* = 0.103) and JOINT (*p* = 0.062), while it is still significant for EXCHANGE. Moreover, neither JOINT (*p* = 0.350) nor EXCHANGE (*p* = 0.119) showed significant improvement compared to IMPULSED. The comparative results of clinical performance across different regression models are shown in Table S3 in the Appendix.

**Table 3:** The clinical performance of microstructural parameters and ADC metrics in identifying rectal lesions with poor- and well/moderate differentiation

Model		AUC	95% CI	Sensitivity	Specificity	Accuracy
Time-dependent ADC	ADC_PGSE_	**0.795**	**0.701–0.889**	**85.7%**	**62.2%**	**75.6%**
	ADC_25Hz_	0.741	0.636–0.845	67.4%	75.7%	70.9%
	ADC_50Hz_	0.732	0.626–0.839	57.1%	81.1%	67.4%
	Multi-ADC	**0.811**	**0.724–0.906**	**87.8%**	**67.6%**	**79.1%**
IMPULSED	${v}_{{in}}$	**0.812**	**0.720–0.905**	**83.7%**	**73.0%**	**79.1%**
	$d$	0.505	0.379–0.631	95.9%	18.9%	62.8%
	${D}_{{ex}}$	0.661	0.542–0.780	69.4%	64.9%	67.4%
	$\rho$	0.764	0.663–0.866	79.6%	64.9%	73.3%
	Combined	**0.847**	**0.762–0.931**	**89.8%**	**70.3%**	**81.4%**
JOINT	${v}_{{in}}$	**0.800**	**0.706–0.894**	**83.7%**	**70.3%**	**77.9%**
	$d$	0.510	0.384–0.636	61.2%	48.7%	55.8%
	${D}_{{ex}}$	0.653	0.534–0.772	75.5%	54.1%	66.3%
	$\rho$	0.747	0.641–0.853	85.7%	56.8%	73.3%
	${k}_{{in}}$	0.740	0.634–0.845	75.5%	70.3%	73.3%
	Combined	**0.869**	**0.792–0.945**	**81.6%**	**86.5%**	**83.7%**
EXCHANGE	${v}_{{in}}$	**0.809**	**0.718–0.900**	**79.6%**	**73.0%**	**76.7%**
	$d$	0.509	0.382–0.635	57.1%	54.1%	55.8%
	${D}_{{ex}}$	0.677	0.562–0.793	69.4%	62.2%	66.3%
	$\rho$	0.760	0.658–0.861	75.5%	70.3%	73.3%
	${k}_{{in}}$	0.780	0.684–0.876	57.1%	89.2%	70.9%
	Combined	**0.883**	**0.810–0.956**	**87.8%**	**78.4%**	**83.7%**

For the calculation of sensitivity, specificity, and accuracy, the classification threshold was determined based on the Youden index, i.e., the feature value corresponding to the maximum Youden index is used as the classification threshold. Note that all regression models are based on the same dataset

*ADC* apparent diffusion coefficient, ${v}_{{in}}$ intracellular volume fraction, $d$ cell diameter, ${D}_{{ex}}$ extracellular diffusivity, $\rho$ image-derived cellularity, ${k}_{{in}}$ water exchange rate constant

For each model, i.e., time-dependent ADC, IMPULSED, JOINT, and EXCHANGE, the results for the single metric with the highest AUC and the combined regression model are highlighted in bold in the table

### The results of pathological section

**Fig. 6:**
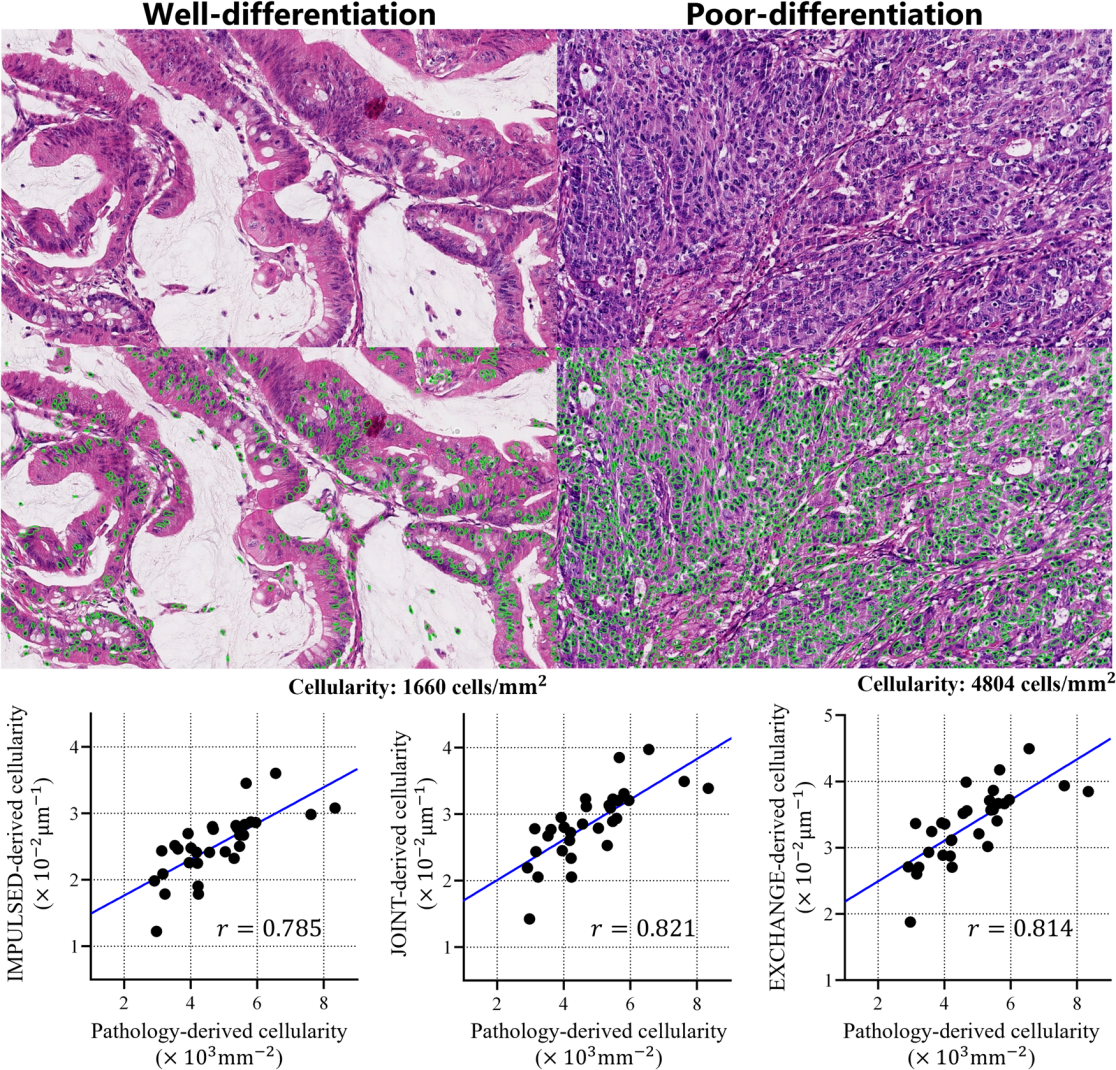
Representative hematoxylin and eosin-stained histopathological morphology (top) shows well-formed glandular structures in a well-differentiated tumor with low cellular density, while in a poorly-differentiated tumor, the glandular architecture is lost, with disorganized cells and high cellular density. The correlation analysis results between radiological and pathological findings for 31 patients (whose H&E-stained sections were available for analysis) are also shown (below). Original magnifications: × 200. The H&E-stained sections originated from the pre-treatment pathological biopsy

Figure 6 demonstrated two representative pathological images after H&E staining and the corresponding results after graphic segmentation. The statistical counts showed that the pathology-derived cell density on the poor-differentiation section was higher than that on the well-differentiation one, which is consistent with the results obtained from MR cytometry imaging, i.e., the cellularity $\rho$ was higher in the poor-differentiation group. Furthermore, cellularity values derived from three MR cytometry methods all exhibited strong positive correlation with the pathology-derived cellularity, with $r$= 0.785, 0.821, and 0.814 for IMPULSED, JOINT, and EXCHANGE, respectively.

## Discussion

This prospective study systematically assessed the clinical performance of MR cytometry in grading the histological differentiation of rectal cancer and compared the fitted results of three different analysis methods. ${{{\rm{AD}}}}{{{{\rm{C}}}}}_{{{{\rm{PGSE}}}}}$ has shown the best diagnostic value among the three different time-dependent ADCs, and the microstructural parameter ${v}_{{in}}$ provided the highest AUC for IMPULSED, JOINT, and EXCHANGE among the classifiers based on a single imaging metric. Compared to the regression model from the traditional single PGSE acquisition (AUC = 0.795), the multivariate regression based on the multi-${t}_{{diff}}$ acquisition protocol and MR cytometry quantitative analysis can significantly improve the final diagnostic accuracy (AUC = 0.847 for IMPULSED, 0.869 for JOINT, and 0.883 for EXCHANGE). This indicated that the joint acquisition of OGSE and PGSE indeed provided additional effective information over a single PGSE sequence. The data analysis combining MR cytometry and ADC measurements outperformed the conventional analysis based solely on the combination of different ADCs (AUC = 0.811 for the multiple ADC model). The above results have demonstrated the clinical potential of additional OGSE acquisitions in rectal cancer diagnosis and MR cytometry analysis in post-processing, despite obvious overlap in imaging metrics across groups.

In this study, the poorly-differentiated rectal cancers showed significantly higher intracellular volume fraction ${v}_{{in}}$ and cellularity $\rho$. One possible explanation could be that these tumors usually exhibit increased nuclear atypia and division patterns, loss of epithelium polarity, disorganization of normal glandular structures and tumor stroma [34]; in contrast, moderately or well-differentiated tumors usually retain larger proportions of normal glandular structures and cell arrangements. In addition, ${v}_{{in}}$ and $\rho$ may be associated with tumor aggressiveness; the higher values reflect the greater aggressiveness and recurrence risk for poorly-differentiated tumors with higher malignancy. This finding aligns with reports in prostate cancer [24] and breast cancer [23, 39, 40], and further supports that MR cytometry may provide similarly satisfactory discriminatory value when assessing lesions in hollow organs as it does in solid organs. Moreover, the cell diameter $d$ exhibited insignificant intergroup difference and poor diagnostic value in identifying differentiation of rectal cancer, which compels us to re-examine this imaging biomarker. In other tumor imaging studies, such as breast [23] and prostate cancer [24], $d$ has also shown poor diagnostic performance. We suppose that ${v}_{{in}}$ and $\rho$ are directly correlated with cell density and proliferation within tumors; in contrast, the model-fitted $d$ represents a coarse average of all cells within an imaging voxel, including tumor, immune, and normal cells, which may be influenced by factors unrelated to the tumor itself, thus limiting its clinical performance.

On the other hand, our results showed that the transcytolemmal water exchange rate constant ${k}_{{in}}$ was significantly lower in poorly-differentiated tumors, which was also consistent with the previous study on the differentiation of benign and malignant breast cancer [23]. We speculated that such lower ${k}_{{in}}$ may be associated with cellular metabolism. Specifically, poorly-differentiated tumor cells primarily rely on anaerobic respiration for metabolism. The shift from aerobic to anaerobic respiration reduces intracellular water molecules or slows their generation, thereby decreasing ${k}_{{in}}$. It should be noted that this is only our hypothesis; further verification through appropriate and intuitive biochemical experiments is necessary. But for imaging-based diagnosis, this finding demonstrates the potential advantage of the additional microstructural parameter ${k}_{{in}}$ in clinics.

Currently, in addition to conventional DWI, ADC measurement, and MR cytometry used in this study, other advanced MR-based imaging techniques were also implemented to achieve the radiological prediction of histological differentiation of rectal cancer, such as virtual magnetic resonance elastography (vMRE) [41], diffusion kurtosis imaging (DKI) [5], and intravoxel incoherent motion (IVIM) imaging [42]. Comparing MR cytometry with these methods is essential to guide and optimize future radiological examinations. Moreover, it also holds clinical significance to integrate results from other morphological MR sequences, such as T1-weighted and T2-weighted images, with MR cytometry imaging, potentially further improving the diagnosis of rectal cancer. We will continue exploring such integration on multi-sequence information in future work. On the other hand, for the diagnosis of rectal cancer and assessment of treatment response, MR cytometry may exhibit synergistic effects with functional imaging methods that reflect tumor metabolic activity, such as ^18^F-Fluorodeoxyglucose (^18^F-FDG) PET-CT [43]. The potential non-monotonic relationship between ^18^F-FDG indicators and MR cytometry results provides an additional approach for revealing the complex correlation between tumor metabolism and microstructure. Integrating these multimodal mappings represents a noteworthy future direction for tumor imaging.

There are several limitations in the current study. First, this prospective study was conducted at a single institution and involved a relatively small sample size, lacking external validation and generalizability. This may explain why JOINT and EXCHANGE failed to achieve significant improvement over IMPULSED, and also accounts for the insignificant improvement in clinical performance when comparing IMPULSED and JOINT to the multi-ADC model, most likely due to the limited sample size. Hence, a larger and multi-center patient cohort is essential for re-validating the clinical findings. Second, the image segmentation analysis on H&E-stained sections failed to cover all enrolled patients. We will collect more H&E-stained pathological sections from rectal cancer patients in future work to achieve a one-to-one comparison between MR cytometry imaging and pathological results. Third, the data analysis in this study was implemented patient-wise or ROI-wise; we only used the mean values of imaging metrics, including microstructural parameters and ADC metrics, across the entire ROI to represent the overall characteristics, neglecting the intra-tumoral heterogeneity, which can be key features of tumor biology and aggressiveness. More heterogeneous information based on MR cytometry results, such as histograms and radiomics features, will be calculated and incorporated into further studies to improve diagnostic value. Furthermore, fixing ${D}_{{in}}$ values in MR cytometry analysis represents another methodological limitation, and the rationality of this approach requires further validation in rectal cancer imaging. Fourth, only patients who had not received any prior treatment were included in this study; mucinous adenocarcinoma and non-surgical cases were also excluded, which limits the generalizability of the clinical findings. We will design more comprehensive inclusion criteria and explore more valuable clinical issues in the future. Finally, although the emerging OGSE sequences have been supported on most 3.0-T MR scanners, they still require further optimization and promotion for implementation on more clinically prevalent 1.5-T scanners. In addition, the current additional 5-min MR cytometry acquisition is acceptable in prospective studies; however, we still need to further shorten the scan time to alleviate the clinical burden and facilitate the integration of this technique into routine MRI examinations. In addition, the current MR cytometry analysis workflow is relatively complex, which poses another significant barrier to the adoption of this emerging technique. We are developing user-friendly post-processing software based on graphical interface operations to facilitate radiologists in model fitting and microstructural parameter quantification.

In summary, this study investigated the clinical performance of emerging MR cytometry imaging and compared different quantitative analysis methods in identifying the differentiation of rectal cancer. The results showed that MR cytometry can provide higher AUC values than conventional DWI and ADC measurements, and the biophysical models incorporating transcytolemmal water exchange, i.e., JOINT and EXCHANGE, outperformed the impermeable IMPULSED model, but the improvement in clinical performance remains insignificant. Future studies will focus on revalidating the current findings on a larger and multi-center patient cohort.

## Supplementary information

Supplementary information

## Data Availability

The datasets used and analyzed during the current study are available from the corresponding author on reasonable request.

## Abbreviations

ADC: Apparent diffusion coefficients
AUC: Area under the curve
$$d$$: Cell diameter
dMRI: Diffusion magnetic resonance imaging
DKI: Diffusion kurtosis imaging
$${D}_{{ex}}$$: Extracellular diffusivity
${D}_{{in}}$: Intracellular intrinsic diffusivity
DWI: Diffusion-weighted imaging
EXCHANGE: Water Exchange, Confined (restricted), and Hindered diffusion under Arbitrary Gradient waveform Encodings
^18^F-FDG: ^18^F-Fluorodeoxyglucose
HR-T2WI: High-resolution T2-wighted image
IMPULSED: Imaging microstructural parameters using limited spectrally edited diffusion
IVIM: Intravoxel incoherent motion
${k}_{{in}}$: Transcytolemmal water exchange rate constant
MRI: Magnetic resonance imaging
OGSE: Oscillating gradient spin-echo
PGSE: Pulsed gradient spin-echo
ROC: Receiver operating characteristic
ROI: Region of interest
$\rho$: Image-derived cellularity
T1WI: T1-weighted image
${v}_{{in}}$: Intracellular volume fraction
Vmre: Virtual magnetic resonance elastography
